# High prevalence of helminth infections in mother-child pairs from three central provinces of Lao People's Democratic Republic

**DOI:** 10.1016/j.parepi.2019.e00122

**Published:** 2019-11-02

**Authors:** Maude Pauly, Kong Sayasinh, Claude P. Muller, Somphou Sayasone, Antony P. Black

**Affiliations:** a Department of Infection and Immunity, Luxembourg Institute of Health, Esch-sur-Alzette, Luxembourg; b Lao Tropical and Public Health Institute, Vientiane, Lao Democratic People's Republic; c Lao-Lux-Laboratory, Institute Pasteur Du Laos, Vientiane, Lao Democratic People's Republic; d Laboratoire National de Santé, Dudelange, Luxembourg

**Keywords:** Parasites, Opisthorchis viverrini, Laos, Liver fluke, Children

## Abstract

In Southeast Asia, the large majority of the population remains affected by parasitic worms despite longstanding mass treatment and health education campaigns. Soil-transmitted helminths and also the fish-borne liver fluke negatively affect development during early childhood.

Here, the prevalence of helminth infections in stool samples of 610 mother-child pairs from Khammouane, Bolikhamxay and Vientiane provinces in Lao People's Democratic Republic was determined by formalin-ethyl acetate concentration technique.

Overall, 15.1% of the children and 46.9% of the mothers were positive for at least one helminth species. Helminth detection rates varied significantly by province with the highest prevelance in Khammouane and the lowest in Bolikhamxay province. Mothers that were positive for soil-transmitted helminths were significantly more likely to have children positive for the same helminth species (p < 0.01) but this was not the case for the liver fluke *Opisthorchis viverrini.* A protective effect of breastfeeding against soil-transmitted helminths was revealed.

Our data reconfirm the generally high helminth burden among mother-child pairs who likely share a number of risky lifestyle behaviors also with other family members. To reduce maternal burden of helminths, we propose that anti-helmintic treatment of women of childbearing age and of mothers during postnatal care should be included in the national strategy.

## Highlights

• Helminth infections are a threat for maternal and child health in Southeast Asia.
• In Lao PDR, the cancerogenic liver fluke, *Opisthorchis viverrini*, is hyper-endemic.
• Risky lifestyle behaviors of mothers and children explain the high helminth burden.
• Soil-transmitted helminths may be indirectly transmitted from mother to child.
• Deworming of women of childbearing age and during postnatal care should be considered.

## 1 Introduction

The control of neglected tropical diseases caused by helminths is challenging. Billions of people in developing countries are affected by these parasitic worms that are associated with childhood anaemia, malnutrition, stunting, and cognitive impairment (Pabalan et al., 2018; Jourdan et al., 2018; Pullan et al., 2014; Hotez et al., 2014). Helminth parasites are mainly transmitted by ingestion of helminthic eggs or larvae that contaminate the environment and food. In addition, larvae of certain helminth species (e.g. hookworm, *Strongyloides stercolaris, etc*) actively penetrate the skin. Hence, inadequate water treatment and sanitation, as well as unsafe nutrition and lifestyle habits are among the major risk factors for infection (Strunz et al., 2014).

As many helminths survive in the environment and have animal reservoirs, a holistic approach is mandatory for their control (Otake et al., 2018; Conlan et al., 2011). Mass deworming campaigns in preschool- and school children have been insufficient, although they reduce the intensity of helminthic infections as shown for Lao children (Phommasack et al., 2008). In Southeast Asia, the vast majority of the population remains affected by parasitic worms despite longstanding mass treatment and health education campaigns (Dunn et al., 2016). For instance, the fish-borne liver fluke, *Opisthorchis viverrini*, remains hyper-endemic in Lao People's Democratic Republic (PDR). Numerous studies underline the public health threat of *O. viverrini* with high infection rates among Lao adults and school-aged children (Sayasone et al., 2007, 2009, 2011; Rim et al., 2003; Saiyachak et al., 2016; Vonghachack et al., 2017; Feldmeier et al., 2016; Forrer et al., 2012). High infection rates among cyprinid fish, traditional consumption of raw fish and sharing of fish dishes among households lead to rapid re-infection after treatment. Also, domestic carnivores may be source of infection for this trematode, but also other zoonotic helminths (Otake et al., 2018; Conlan et al., 2011, 2012; Vonghachack et al., 2017).

In Lao PDR, approximately 30% of the population still have no access to safe drinking water and adequate sanitation. Open defecation is still common practice for an estimated 23% of the population (UNDP, 2019). One consequence is certainly high rates of infection with soil-transmitted helminths (STH) including *Ascaris lumbricoides*, *Trichuris trichiura* and hookworms (*Ancylostoma duodenale, Necator americanus and Ancylostoma ceylanicum*) (Dunn et al., 2016).

Mass deworming with the anthelmintic drugs, mebendazole or albendazole, target mainly school-aged children and only rarely pre-school children or adults. However children are often already infected at an early age. In Lao PDR, mebendazole is administered to children during deworming campaigns. From other tropical regions, it was reported that maternal infections with STH were associated with an increased risk of infection for children (Menzies et al., 2014; Mehta et al., 2012). Hence, deworming of the mothers during postnatal care and possibly also during antenatal care (after the first trimester of pregnancy) is considered a sound approach to improve the health of both the mother and the child (Mofid and Gyorkos, 2017; Salam et al., 2015). While a single dose of mebendazole or albendazole is effective to reduce the overall STH burden, it is ineffective against liver fluke. Praziquantel is the drug of choice for treating of *O. viverrini*. However, a recent study suggested that Tribendimidine could represent a potential alternative treatment with less adverse events (Sayasone et al., 2018). In this study, we assessed the prevalence of helminth infection among mother-child pairs and investigated whether Lao mothers and their children have a similar risk to be infected with *O. viverrini,* as well as other helminth species.

## 2 Materials and methods

Between December 2013 and July 2014, 610 paired mother-child serum and stool samples were collected in the framework of an unrelated serological vaccine response study in three provinces (Bolikhamxay, Khammouane, Vientiane) of Lao PDR (Evdokimov et al., 2017). Stool samples (2 g) were collected into 10% fomalin and screened for helminth parasite using formalin-ethyl acetate concentration technique (Cociancic et al., 2018; Elkins et al., 1991) Helminth species were identified by light microscopy with a magnification of 400 based only on morphological characteristics (i.e. size and shape) of eggs. Parasite species was not confirmed with molecular methods. *O. viverrini* eggs were differentiated from those of Minute Intestinal Flukes by demonstrating the distinct morphological characteristics, e.g., shoulders at operculum, eggshell and knob. It was not possible to further different the species of Minute Intestinal Flukes by this technique. Helminth positivity was defined as the presence of at least one helminth ovum in the stool sample, STH positivity as the presence of at least one STH ovum in the stool sample. As helminth eggs were not counted, infection intensity could not be evaluated. The study was approved by the Lao National Ethics Committee (NECHR860).

The median age of the mothers was 27.0 years (age range: 17–49 years) and most had a normal Body Mass Index (BMI). The median age of the children was 20.6 months (age range: 9.2–49.8 months) and the majority had a good nutritional status as determined by BMI, as well as other anthropometric indicators of childhood nutritional status (i.e. weight-for-height, height-for-age, weight-for-age, and Mid-Upper Arm Circumference z-scores) (see Table 1). While more than 98% of the children were breastfed, breastfeeding duration ranged from 0 to 49 months (mean: 11.1 months) and 80% of the children were breastfed for less than 6 months or not at all. Information on gender and birthplace were obtained and the maternal and child antibody status (presence or absence of antibodies against Diptheria, Tetanus, Measles, Rubella and Hepatitis B) was retrieved from a previous study (Evdokimov et al., 2017).

**Table 1:** Socio-demographic characteristics of participants.

Cohort	Factors		n (%)
Women	Age group (years)	<25	182 (30.8)
		25–30	247 (41.9)
		>30	161 (27.3)
		not available	20
	Body mass index	<18.5	84 (14.2)
		18.5–22.9	299 (50.7)
		>23	220 (37.3)
		not available	7
Children	Gender	Female	325 (53.3)
		Male	285 (46.7)
	Age group (months)	≤24	394 (64.8)
		>24	214 (35.2)
	Body mass index (z-scores)	<-2	37 (6.2)
		≥-2	562 (93.8)
		not available	11
	Birthplace	home or health-center	233 (38.4)
		hospital	374 (61.6)
		not available	3
Mother-child pairs	Province	Bolikhamxay	143 (23.4)
		Khammouane	287 (47.0)
		Vientiane	180 (29.5)
	Total		610 (100)

Statistical analyses were performed in R software (version 3.1.0.; R Foundation for Statistical Computing, Vienna, Austria [https://www.r-project.org/]). Two-sided Fisher's exact or chi-squared tests were applied to identify factors associated with helminth parasite infections in mothers and children: province, age group, nutritional status, birthplace, antibody status and coinfection. In addition, the association between the parasite burden and the antibody status was assessed. A p-value of 0.05 was used as the cutoff for significance .

## 3 Results

Overall, 15.1% (92/610) of the children and 46.9% (286/610) of the mothers were positive for at least one helminth species. STH (i.e. *A. lumbricoides*, *T. trichiura* and hookworms) accounted for about half of the helminths detected in the children stool samples, but only 31.8% of the helminths in the maternal samples (Table 2). The trematode *O. viverrini* was the most prevalent helminth overall among both children and mothers and was detected in 7.5% and 37.7% of samples, respectively. Consequently, 46 out of the 92 positive children and 230 out of the 286 positive mothers were positive for this trematode. While *A. lumbricoides* was the most prevalent STH in children (54.5%; 24/44), hookworms represented 64.8% (56/91) STH detected in the mothers. Besides, eggs of hookworm*, T. trichiura* and Minute Intestinal Flukes were detected in 2.0%, 2.1% and 1.3% of the children and in 9.2%, 5.1% and 6.4% of the mothers (Table 2). While 13% of the children were positive for more than one helminth species, this was the case for 28.3% of the mothers. In most cases, eggs of different STH species or of one STH species and of *O. viverrini* were detected. Many mothers were also positive for the 2 Trematoda species*, O. viverrini* and Minute Intestinal Flukes.

**Table 2:** Helminth positivity among children and women.

Class	Genus	Children		Women	
		n (%_Positivity_^b^)	%_Relation_^c^	n (%_Positivity_^b^)	%_Relation_^c^
Nematoda	Hookworm	12 (2.0)	13.0	56 (9.2)	19.6
	*Ascaris lumbricoides*	24 (3.9)	26.1	24 (3.9)	8.4
	*Enterobius vermicularis*	1 (0.2)	1.1	1 (0.2)	0.3
	*Strongyloides stercoralis*	1 (0.2)	1.1	3 (0.5)	1.0
	*Trichuris trichiura*	13 (2.1)	14.1	31 (5.1)	10.8
Trematoda	Minute Intestinal Flukes	8 (1.3)	8.7	39 (6.4)	13.6
	*Opisthorchis viverrini*	46 (7.5)	50.0	230 (37.7)	80.4
Cestoda	*Taenia* spp.	0 (0)	0.0	3 (0.5)	1.0
STH^a^		44 (7.2)	47.8	91 (14.9)	31.8
All helminths		92 (15.1)	100.0	286 (46.9)	100.0

a Soil-transmitted helminths: Hookworm (*Ancylostoma*), *Trichuris trichiura* and *Ascaris lumbricoides*.

b positivity rate of specific helminth in total samples.

c positivity rate of specific helminth in relation to the overall helminth positivity.

**Fig. 1:**
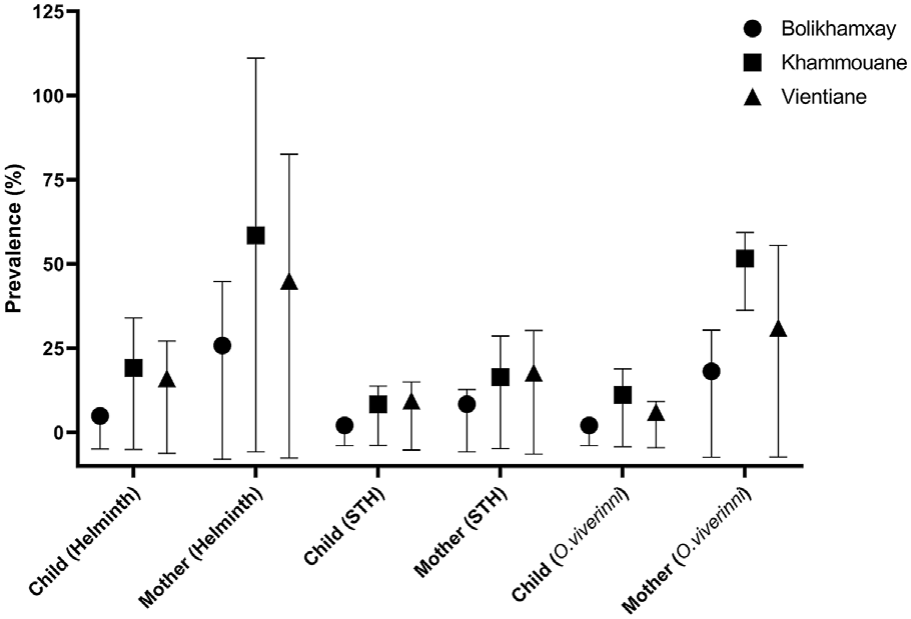
*Prevalence of helminth infections among children and women in 3 Lao provinces.* The graph represents the helminth prevalance among children and mothers in Vientiane, Khammouane and Bolikhamxay provinces. “STH” = Soil-transmitted helminths including Hookworm (*Ancylostoma*), *Trichuris trichiura* and *Ascaris lumbricoides*. “Helminths” refers to all helminthic species detected in the samples and comprises thus STH, *Opisthorchis viverrini*, and other species.

Helminth positivity varied significantly by province (Fig. 1). While helminth positivity was established for 58.5/19.2% of the mother-child pairs in Khammouane province, this rate was 45.0%/16.1% in Vientiane province and 25.9/5.6% in Bolikhamxay province. In contrast, similar STH positivity rates were found in Vientiane and Khammouane provinces (17.8/9.4% and 16.4/8.4%), but again the lowest rate was found in Bolikhamxay province (8.4/2.1%). Also for *O. viverrini*, positivity rates were lowest in Bolikhamxay province (18.2/2.1%), highest in Khammouane province (51.6/11.2%) and intermediate in Vientiane province (31.1/6.1%). Children that were breastfed less than 6 months were significantly more likely to be positive for STH (p = 0.021; OR = 2.5 [1.2–5.1]), whereas duration of breastfeeding had no statistically significant effect on the odds of *O. viverrini* positivity. Birthplace and gender did not influence the helminth or STH status. Also, there was no statistically significant association between the helminth or STH status and nutritional status or antibodies against diptheria, tetanus, measles, rubella or hepatitis B.

For 62.0% of the positive children, their mothers were also positive for at least one helminth (p < 0.01). When only the STH were taken into account, this was the case for 52.3% of the positive children (p < 0.01): mothers that were positive for *Ancylostoma, Ascaris* or *Trichuris* were significantly more likely to have children positive for the same helminths (p < 0.01). While 50% of the children positive for *O. viverrini* had mothers who were positive for the same trematode, this association was not statistically significant (Table 3).

**Table 3:** Prevalence of concurrent helminth infections among mother-child pairs.

	Concurrent helminth infections among mother-child pairs		
	n (%^a^)	p-value	OR [95% CI]^b^
Helminths	57 (9.3)	0.002	2.1 [1.3–3.2]
Soil-transmitted helminths	23 (3.8)	<0.001	8.0 [4.2–15.3]
Hookworms	6 (1.0)	<0.001	10.9 [3.4–35.2]
*Ascaris lumbricoides*	9 (1.5)	<0.001	22.8 [8.6–60.4]
*Trichuris trichiura*	4 (0.7)	0.003	9.4 [2.7–32.4]
*Opisthorchis viverrini*	23 (3.8)	0.083	1.72 [0.9–3.3]

a rate (n/N*100) of concurrent helminth infections among all mother-child pairs, where N = 610.

b Odds Ratio [95 percent confidence interval].

## 4 Discussion

Lao PDR has a dramatically high rate of child malnutrition due to inappropriate nutrition practices (The state of the, 2016) but also due to the high helminth burden. Socioeconomic factors such as lack of safe water sources and poor sanitation facilitate the spread and complicate the control of helminths. We show here that a high proportion of preschool children in Bolikhamxay, Khammouane and Vientiane provinces get a helminthic infection already during early childhood.

Overall 15.1% (92/610) of the children and 46.9% (286/610) of the mothers were positive for at least one helminth, with *O. viverrini* being the most prevalent in each province. High positivity rates were reported already for preschcool children from Savannakhet province (Kounnavong et al., 2011), Champasack province (Sayasone et al., 2011) and Huaphan province (Nanthavong et al., 2017). Similar to the latter study (Nanthavong et al., 2017), we could not find a significant association between nutritional status and positivity for STH or *O. viverrini*. Nevertheless, the health consequences of the high helminth burden may be dramatic. *O. viverrini* is one of the main drivers of cholangiocarcinoma, a cancer of the biliary duct system. It was shown that repeated reinfections and treatments may accelerate carcinogenesis in addition to smoking, the consumption of alcohol and fermented meats and infection with hepatitis B and/or C virus (Steele et al., 2018). In line with a previous study reporting that a high proportion of patients in Vientiane and Khammouane provinces had liver lesions suggestive of this serious cancer (Kim et al., 2018), we find the highest positivity rates of *O. viverrini* in these same two provinces. This can likely be explained by high rates of under-cooked fish consumption as numerous streams and rivers flow through Khammouane province and as one of the country's largest lakes, Nam Ngum, is situated in Vientiane province. Previously, the probable association between proximity of the Mekong River and helminth infection was shown for primary schoolchildren (Rim et al., 2003).

The overall helminth positivity rate was lower than in previous studies. This may be explained by a certain sampling bias. The dataset includes only fully vaccinated children. In contrast to Lao children from remote mountainous areas, these children have had access to health care and probably already received an anthelminthic treatment. Nevertheless, this study provides further evidence that mass deworming campaigns using mebendazol (mebendazole 500 mg tablet, Johnson & Johnson, New Jersey, USA) and targeting only children are insufficient for STH control and are ineffective against *O. viverrini*. We found that mothers infected with STH are significantly more likely to have children infected with the same parasite. Whether this reflects similar environmental and dietary exposure to helminth eggs remains unclear.

Interestingly, our study also revealed that children that were breastfed for at least 6 months were less likely to be infected with STH. However, breastfeeding had no protective effect against *O. viverrini* and, mothers infected with *O. viverrini* were not significantly more likely to have children infected with the same parasite despite the overall high prevalence. This latter finding contradicts a recent study conducted in Khammouane province among mothers and their children aged 5–15 years (Araki et al., 2018). It is known that many mothers do not exclusively breastfeed their infant during the first 6 months (Lee et al., 2013). Here, mothers were only asked whether and for how long their infant was breastfed. Unfortunately, the questionnaire did not record any information about complemental food. Lao postpartum practices include feeding infants with chewed glutinous rice, water (Barennes et al., 2009) and coffee creamer (Barennes et al., 2008). This complemental food may be a source of STH, but not of *O. viverrini* infections during early childhood. In fact, insufficient hygiene practices when preparing food or when consuming uncooked foods contaminated with parasite eggs may explain the overall high STH positivity observed among mother/child pairs. We suspect that the diet of children and mothers differs the first five years after birth, but coincides as the child grows older. At age 5–15 years, dietary risk factors for *O. viverrini* (e.g. consumption of raw fish dishes) are likely shared by child and mother.

This study shows that mother-child pairs are similarly exposed to STH. This is likely due to shared risk factors. Consequently, mass deworming campaigns should, in future, also include the parents. In view of the high maternal positivity rates for *O. viverrini*, we suggest that targeted treatment of women and teenage girls and in particular of mothers during postnatal care should be included in the national strategy towards the control of this trematode and of STH (Mofid and Gyorkos, 2017; Gyorkos et al., 2018). At this timepoint, the benefits of breastfeeding and of other effective measures for the prevention of helminth infections, as well as the inherent risk associated to repeated reinfections and treatments could also be comprehensively explained. Finally, a One Health approach is essential for assuring a sustainable control of zoonotic helminths in Southeast Asia.

## Declarations of interest

None.
